# Interactive effects of irrigation and nitrogen management on greenhouse gas emissions and resource efficiency in alfalfa production

**DOI:** 10.3389/fpls.2025.1740107

**Published:** 2026-01-22

**Authors:** Tianyi Qu, Jiabei Li, Xiaodan Song, Xiaobo Luan, Jie Pang, Shikun Sun, Yubao Wang

**Affiliations:** 1 College of Water Resources and Architectural Engineering, Northwest A&F University, Yangling, Shanxi, China; 2 Key Laboratory of Agricultural Soil and Water Engineering in Arid and Semiarid Areas, Ministry of Education, Northwest A&F University, Yangling, Shaanxi, China

**Keywords:** crop yield, GHG emissions, global warming potential, *Medicago sativa* L, sustainable management, water-nitrogen interactions

## Abstract

**Introduction:**

Mitigating agricultural greenhouse gas (GHG) emissions while maintaining forage productivity is a key challenge under global carbon-neutrality goals. To evaluate the environmental and agronomic trade-offs of irrigation and nitrogen management, a field experiment was conducted in an arid region of Northwest China.

**Methods:**

Twelve irrigation-nitrogen treatment combinations were applied to alfalfa (Medicago sativa L.) to quantify N_2_O, CO_2_, and CH_4_ fluxes, global warming potential (GWP), and resource-use efficiencies.

**Results:**

Results showed that soil water-filled pore space and available nitrogen strongly regulated N2O, emissions, with peaks occurring within one week after irrigation or fertilization. Excessive water and nitrogen inputs significantly increased GHG emissions and reduced irrigation water productivity (IWP) and partial factor productivity of nitrogen (PFPN).

**Discussion and Conclusion:**

Conversely, the high-water, moderate-nitrogen regime (300 mm irrigation + 120 kg N ha^-1^) achieved a balanced outcome—sustaining high yield while reducing cumulative N_2_O emissions by 29.5-93%, total GWP (LCA-based) by 24.1%, and greenhouse gas emission intensity (GHGI) by 29.0% relative to conventional high-input management (W2N3). These preliminary findings suggest a water-nitrogen synergy zone that improves yield-GHG trade-offs, though multi-year validation is required.

## 1 Introduction

Global warming has emerged as one of the world’s most pressing environmental challenges. Given the growing international interest in sustainable development, many nations have set targets to achieve carbon neutrality by the second half of the 21st century (Pawłowski and Rydzewski, 2024). Agricultural ecosystems are significant sources of greenhouse gases (GHGs), contributing 10–12% of global emissions (Shukla et al., 2022). Grasslands, a vital component of agricultural ecosystems, provide critical ecological functions. In particular, grasslands play a crucial role in biodiversity conservation, soil and water management, and regulation of carbon and nitrogen cycles (Wright, 2006). Both forage security and environmental sustainability must be addressed in order to achieve sustainable agricultural development with limited land resources under global climate change.

Irrigation and nitrogen management can improve crop growth and yield (Wang et al., 2024), impacting agricultural productivity and GHG emissions from grasslands. Under optimal irrigation and fertilization practices, water and nitrogen uptake by plant roots is promoted, leading to enhanced resource use efficiency and reduced soil GHG fluxes; as such, higher crop yield and lower GHG emissions are achieved. However, in arid and semi-arid regions, irrigation substantially boosts crop yield while increasing soil GHG emissions, particularly N_2_O and CO_2_ fluxes (Qin et al., 2024; Tan et al., 2025). Moreover, nitrogen fertilizers applied to soil are primarily lost through irrigation-induced leaching (NO_3_^–^) and microbial-mediated emissions (N_2_O, NO, NH_3_), exceeding crop uptake and causing environmental issues (Yoon et al., 2019). In particular, intensive water and nitrogen management can significantly increase N_2_O emissions. For instance, emission factors observed in intensive systems can exceed the Intergovernmental Panel on Climate Change (IPCC) default value of 1.0%, highlighting the need for region-specific assessments. Conversely, high irrigation levels (>120 mm) decrease CH_4_ oxidation rates by 42–79% due to enhanced pore water saturation of soil (Ning et al., 2022; Zhang et al., 2017a).

Alfalfa (*Medicago sativa* L.) is a widely cultivated and highly nutritious fodder plant, known as the “king of forage crops.” Alfalfa has an excellent capacity for biological nitrogen fixation through root nodules, reducing its need for nitrogen fertilizers (Robertson et al., 2000). However, low nitrogen rates limit root nodule development, which in turn restricts nitrogen supply to leguminous crops. Rational nitrogen management influences interactions between legume root exudates and soil microbes, altering carbon and nitrogen cycles and consequently affecting soil GHG emissions (Du et al., 2024; Zong et al., 2025). Under water-saturated conditions, denitrification in the crop rhizosphere can cause localized surges in N_2_O emissions (Zhang et al., 2021). The combination of irrigation and nitrogen application exacerbates GHG emissions in intensive agricultural ecosystems (Qu et al., 2025). Thus, optimizing irrigation and fertilization regimes to balance crop productivity and emission reductions—by adjusting soil moisture and nitrogen conditions—is crucial for alfalfa production.

The lock-in effects of “water-nitrogen inputs – yield gains – GHG emission reductions” are complex in grasslands. For example, in the arid region of northwest China, limiting nitrogen application (150–200 kg ha^-^¹) with drip irrigation (≤360 mm) can increase alfalfa hay yield by 18–24% and reduce global warming potential (GWP) per unit yield by 31% compared to conventional management (Kamran et al., 2022; Ning et al., 2022). However, several aspects remain less explored. First, single-factor or narrow-range designs still dominate, so water–nitrogen interactions across a full management gradient are not yet fully resolved. Second, most campaigns rely on low-frequency GHG sampling, which tends to overlook short-term emission pulses triggered by irrigation or fertilization events. Third, simultaneous consideration of forage productivity, water- and nitrogen-use efficiencies, and non- CO_2_ greenhouse gas emissions—needed to quantify production–environment trade-offs—is still scarce. To address these limitations, we applied a 3×4 factorial water–nitrogen experiment coupled with high-frequency flux measurements to capture both seasonal patterns and event-driven peaks. By jointly analyzing yield, irrigation water productivity (IWP), partial factor productivity of nitrogen (PFPN), GWP and greenhouse gas emission intensity (GHGI), we evaluate the possibility of identifying a site- and year-specific water–nitrogen synergy zone under the semi-arid conditions of north-western China. This demonstrates the feasibility of maintaining crop productivity and mitigating GHG emissions through optimized irrigation and nitrogen application.

The aims of the present study were to: (a) quantify soil GHG emissions over the alfalfa growing season under different water-nitrogen regimes; (b) determine the GWP and GHGI in alfalfa systems under varying water-nitrogen conditions; and (c) examine the impacts of water-nitrogen treatments on resource use efficiency in alfalfa. A multi-gradient water-nitrogen coupling experiment was conducted in the arid region of Northwest China to investigate the response mechanisms of soil GHG emissions from alfalfa fields and quantify their GWP. The results could contribute to understanding of the greenhouse effect arising from water-nitrogen management and providing support for low-carbon agriculture.

## 2 Materials and methods

### 2.1 Experimental site

**Figure 1:**
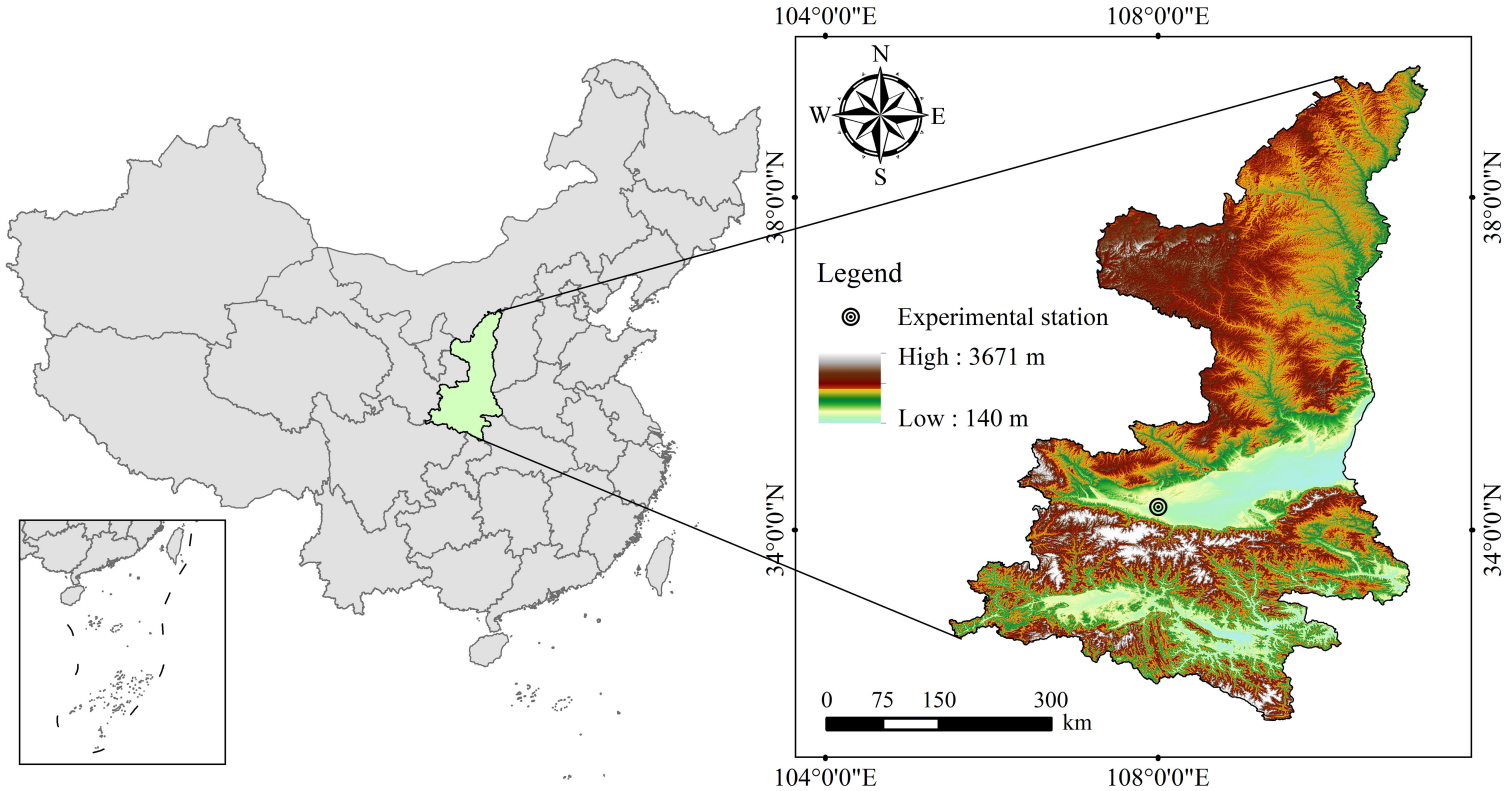
An overview map of the study area in Yangling, Shaanxi Province, China.

**Figure 2:**
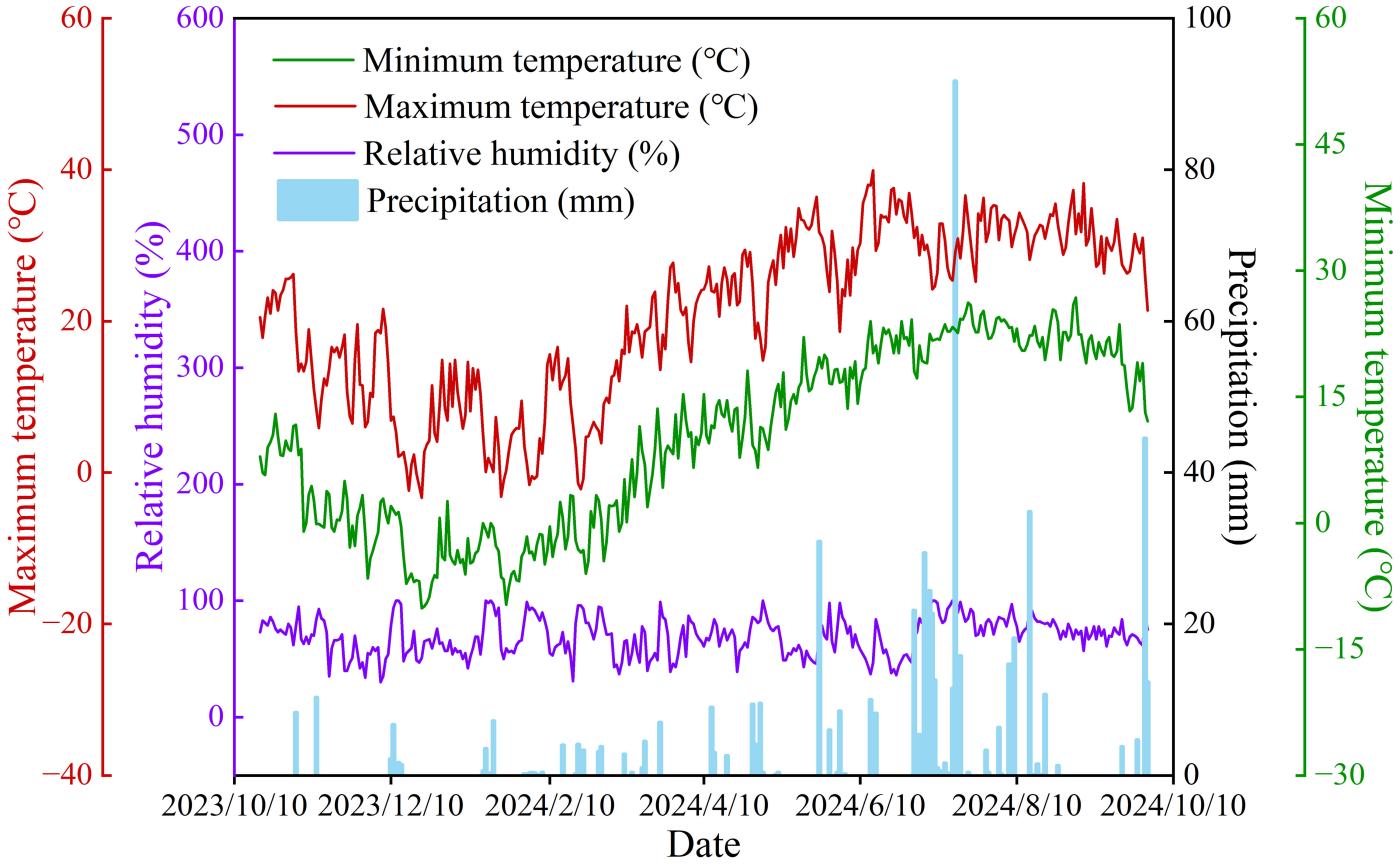
Daily temperature and precipitation over the alfalfa growing season (2023–2024).

The field experiment was conducted at the Water-Saving Irrigation Experimental Station, Key Laboratory of Agricultural Soil and Water Engineering in Arid and Semiarid Areas (Ministry of Education), Northwest A&F University. The experimental site is located in the Yangling Demonstration Zone, Xianyang, Shaanxi Province, Northwest China (108°24’ E, 34°20’ N; **Figure 1**). This area is situated in the western part of the Guanzhong Plain with a long-term average annual temperature of 12.9 °C. The long-term mean annual precipitation at the study site is 632 mm (1981–2020 climatology), whereas the experimental year (2023–2024) received 578.7 mm of precipitation according to observations from the on-site meteorological station. To ensure realistic soil-moisture scenarios, the three irrigation levels (W0, W1, W2) were designed after analyzing the 1981–2020 rainfall distribution: about 70% of annual precipitation falls between June and September, often as high-intensity events ≥ 10 mm day^-^¹. The irrigation schedules therefore mimicked dry-year (W0), average-year (W1) and wet-year (W2) moisture regimes by supplementing 0, 100 and 300 mm, respectively, during the spring and early-summer gaps when alfalfa root uptake is highest. The timing and quantity of irrigation were therefore aligned with the seasonal rain. Precipitation and temperature data for the study area in the experimental period were obtained from a meteorological station within the experimental site (**Figure 2**). The soil type is classified as Haplic Cambisol characterized by a clay-loam texture and a field capacity of ~22.4% (gravimetric moisture content) in the 0–20 cm surface layer. The basic physicochemical properties of the surface soil were measured prior to sowing: pH (potentiometric method), 8.5; total nitrogen (Kjeldahl method), 1.0 g kg^-^¹; available phosphorus (Olsen method), 15 mg kg^-^¹; nitrate nitrogen (continuous flow analysis), 23 mg kg^-^¹; ammonium nitrogen (continuous flow analysis), 8 mg kg^-^¹; total organic matter (dichromate oxidation method, Walkley-Black), 15.5 g kg^-^¹; and bulk density (cutting ring method), 1.48 g cm^-^³.

### 2.2 Experimental design

The experiment was carried out between October 2023 and October 2024. To ensure that the experimental design aligns with local agronomic practices, the irrigation and fertilization levels were established based on regional water availability and the actual water and nitrogen requirements of alfalfa in the study area. The Yangling region has an average annual precipitation of approximately 630 mm, mostly occurring from June to September. The irrigation levels (W0: 0 mm, W1: 100 mm, W2: 300 mm) were all within the conventional supplemental irrigation range for local alfalfa production (100–350 mm). The irrigation treatments were therefore designed primarily for comparison purposes under different water conditions in relation to greenhouse gas (GHG) emissions and yield performance.

The nitrogen application rates (N0: 0 kg ha^-^¹, N1: 60 kg ha^-^¹, N2: 120 kg ha^-^¹, N3: 180 kg ha^-^¹) were based on the locally recommended fertilization range for alfalfa (60–180 kg ha^-^¹), covering low to high levels typically observed in production. Such a design provides regionally applicable insights into the balance between agronomic productivity and environmental sustainability under variable water–fertilizer regimes. A total of 12 water-nitrogen management schemes were established (**Table 1**), with each replicated three times across 36 plots. The plots (2 m × 2 m each) were separated by 1 m-wide isolation strips and furrows. Seeds of the alfalfa variety WL63HQ (Autumn Sleep Grade 5) were obtained from Zhengdao Seed Industry Co., Ltd. (Beijing, China) and sown at a rate of 25 kg ha^-^¹ with a row spacing of 20 cm. The fertilizers used in the experiment included superphosphate (46% P_2_O_5_, 75 kg ha^-^¹), potassium fertilizer as K_2_O (90 kg ha^-^¹), and urea (46% N). Urea (46% N) were applied with a basal-to-topdressing ratio of 4:6. Basal nitrogen fertilizer (40%) was applied at sowing on October 15, 2023, followed by two topdressings (30% each) on March 14 (first harvest) and June 4 (second harvest), 2024. Irrigation took place on March 14 and April 14 (first harvest), June 4 (second harvest), and August 11 (third harvest), 2024. Alfalfa crops were mowed at the initial flowering stage, with three harvests scheduled annually on May 15, July 20, and September 27, 2024. Standard agronomic practices were maintained consistently across all treatments to isolate the effects of water and nitrogen. Prior to sowing, rotary tillage was performed to prepare a uniform seedbed. Basal fertilizers (Phosphate and Potassium) were incorporated into the soil during tillage to ensure adequate nutrient supply. During the growing season, integrated pest and weed management (using standard insecticides and manual weeding) was applied uniformly when necessary to prevent biotic stress. Harvest operations were conducted mechanically at a stubble height of 3 cm.

**Table 1:** Irrigation and nitrogen management for the alfalfa growing season in 2023–2024.

Treatment	Irrigation volume (mm)			Total irrigation volume (mm)	Nitrogen rate (kg ha^-^¹)
	First harvest	Second harvest	Third harvest		
W0N0	0	0	0	0	0
W0N1	0	0	0	0	60
W0N2	0	0	0	0	120
W0N3	0	0	0	0	180
W1N0	40	30	30	100	0
W1N1	40	30	30	100	60
W1N2	40	30	30	100	120
W1N3	40	30	30	100	180
W2N0	120	90	90	300	0
W2N1	120	90	90	300	60
W2N2	120	90	90	300	120
W2N3	120	90	90	300	180

### 2.3 Gas sampling and analysis

#### 2.3.1 Field sampling and measurements

Soil GHG emissions are primarily concentrated in spring, summer, and autumn (Yang et al., 2025). Therefore, gas sampling was conducted from the first regreening stage (March 13, 2024) to the last harvest (September 28, 2024) of alfalfa using a static dark chamber method, and gas chromatography was adopted to measure N_2_O, CO_2_, and CH_4_ fluxes in field plots (Ning et al., 2020). The sampling chamber was made of stainless steel, consisting of a top chamber (30 cm × 30 cm × 30 cm) and a base (filled with water in a 3 cm-deep trough to seal the chamber). The chamber was placed at the center of each plot, with the base edge level with the ground surface. After sealing the chamber, a 30-ml syringe was used to collect gas samples at 0, 10, 20, and 30 min, resulting in four samples per treatment. Soil temperature (0–5 cm) within the chamber was measured during sampling. Gas samples were collected every 7–10 days between 9:00 and 11:00 AM. After irrigation and fertilization, consecutive alternate-day sampling was initiated on day 1 and continued for 7–10 days. Additional measurements were taken after heavy rainfall (>10 mm).

#### 2.3.2 Calculation of GHG emission fluxes

The peak area of the sample gas was measured using an Agilent 7890B gas chromatograph (Santa Clara, CA, USA). The gas concentration was calculated from the peak area using Equation 1:

(1) $$\begin{array}{c} C_{S}=\frac{A_{S}\times C_{0}}{A_{0}} \end{array}$$

where *C_S_* represents the concentration of the target gas in a given sample (ppm, ppb); *A_S_* is the peak area of the sample gas; *C_0_* is the certified concentration of the standard gas (N_2_O: 350 μL m^-^³, CO_2_: 500 mL m^-^³, CH_4_: 2 μL m^-^³); and *A_0_* is the peak area of the standard gas.

The GHG emission flux at the sampling time was determined based on the linear regression slope of gas concentration in four consecutive samples per treatment using Equation 2 (Liu et al., 2017):

(2) $$\begin{array}{c} F=\frac{M}{22.4}\times \frac{273}{273+T_{a}}\times 60\times H\times \frac{d_{C}}{d_{t}} \end{array}$$

where *F* is the gas flux (N_2_O, CH_4_: μg m^-^² h^-^¹; CO_2_: mg m^-^² h^-^¹); *M* is molar mass (g mol^-^¹); *T_a_* is chamber air temperature (°C); *H* is chamber height (m); and *dC/dt* is the concentration change rate (μL L^-^¹ min^-^¹ for CO_2_; nL L^-^¹ min^-^¹ for N_2_O and CH_4_). Eq. (2) assumes ideal-gas conditions at 1 atm and *T_a_;*.

#### 2.3.3 Quantification of cumulative GHG emissions

The cumulative GHG emissions over the alfalfa growing season were calculated using Equation 3 (Zhang et al., 2021):

(3) $$\begin{array}{c} GF=\sum_{i=1}^{n}\frac{F_{i+1}+F_{i}}{2}\times t_{i+1}-t_{i}\times 24\times \kappa \end{array}$$

where *GF* denotes the cumulative emission of the measured gas (N_2_O: kg N ha^-^¹, CO_2_ and CH_4_: kg C ha^-^¹); *F_i_* is the gas emission flux at the *i*-th sampling time (N_2_O and CH_4_: μg m^-^² h^-^¹; CO_2_: mg m^-^² h^-^¹; (*t_i+1_−t_i_*) is the time interval between two consecutive measurements (days); *K* is the unit conversion factor (N_2_O and CH_4_: 10^–5^, CO_2_: 10^–2^); and *n* is the total number of measurements.

### 2.4 Soil and forage sampling and analysis

#### 2.4.1 Determination of soil properties

Soil sampling was conducted at the same time of gas sampling throughout the experiment. After each gas collection, soil temperature at a 5 cm depth was recorded outside the chamber using a soil thermometer. Then, soil samples were taken from the 0–20 cm depth using a 5-cm diameter soil auger, and the moisture content was determined using the oven-drying method. Available nitrogen was extracted with 1 mol/L KCl at a soil-to-solution ratio of 1:10 (*w/v*), and quantification of NO_3_^–^ and NH_4_^+^ contents was conducted using a SEAL AA3 continuous flow analyzer (Norderstedt, Germany).

Water-filled pore space (WFPS) was calculated using Equation 4 based on the method of Bergsma et al. (2002):

(4) $$\begin{array}{c} WFPS=(\frac{\theta }{1-\frac{\rho_{b}}{\rho_{p}}})\times 100 \end{array}$$

where *WFPS* denotes the water-filled pore space (%); *θ* is the volumetric moisture content determined using the cutting ring method (%); *ρ_b_* is the soil bulk density (g cm^-^³); and *ρ_p_* is the particle density (typically assumed to be 2.65 g cm^-^³).

#### 2.4.2 Evaluation of forage yield and resource use efficiency

Three 50 cm × 50 cm quadrats were established in each field plot. During the initial flowering stage, alfalfa crops were harvested at a 3-cm stubble height and transported to the laboratory for oven drying. Forage samples were first inactivated at 105°C for 15 min and then dried at 65°C until constant weight. Alfalfa hay yield for each treatment was calculated and converted to a per-hectare basis (kg ha^-^¹).

Irrigation water productivity (IWP) was obtained as the ratio of alfalfa hay yield to total irrigation volume using Equation 5 (Kamran et al., 2022), and partial factor productivity of nitrogen (PFPN) was calculated as the ratio of alfalfa hay yield to nitrogen rate using Equation 6 (Kamran et al., 2022):

(5) $$\begin{array}{c} IWP=\frac{Y}{I} \end{array}$$

(6) $$\begin{array}{c} PFPN=\frac{Y}{N} \end{array}$$

where *IWP* is the irrigation water productivity, defined as the ratio of alfalfa hay yield (kg ha^-^¹) to total irrigation water applied (m³ ha^-^¹), with units of kg m^-^³ (i.e., kilograms of dry yield per cubic meter of irrigation water). *PFPN* is the partial factor productivity of nitrogen (kg kg^-^¹), *Y* is the alfalfa hay yield (kg ha^-^¹), *I* is the total irrigation volume (m³ ha^-^¹), and *N* is the nitrogen rate (kg N ha^-^¹).

### 2.5 Estimation of N_2_O emission factor, GWP, and GHGI

The N_2_O emission factor (EF) was estimated using Equation 7 (IPCC, 2014):

(7) $$\begin{array}{c} EF=\frac{GF_{F}-GF_{N}}{N_{F}}\times 100 \end{array}$$

where *EF* represents the N_2_O emission factor (%); *GF_F_* and *GF_N_* are the cumulative N_2_O emissions from fertilized and unfertilized treatments, respectively (kg N ha^-^¹); and *N_F_* is the nitrogen rate (kg N ha^-^¹).

To comprehensively evaluate the environmental impact of the alfalfa production system, the net global warming potential (GWP, kg CO_2_-eq ha^-^¹) was calculated using a Life Cycle Assessment (LCA) approach. This method integrates both direct soil GHG emissions and indirect emissions associated with agricultural inputs (fertilizer production, irrigation electricity, and diesel fuel). The calculation is expressed as shown in Equations 8 and 9:

(8) $$\begin{array}{c} GWP=GWP_{soil}+GWP_{inputs} \end{array}$$

(9) $$\begin{array}{c} GWP_{inputs}=\sum \left(Amount_{i}\times EF_{i}\right) \end{array}$$

where $GWP_{inputs}$ represents the *GWP* from agricultural inputs (kg CO_2_-eq ha^-^¹). The carbon emission factors ( $EF_{i}$) were adopted from recent literature on Chinese agriculture: 8.3 kg CO_2_-eq kg^-1^ for N fertilizer production, 0.79 kg CO_2_-eq kg^-1^ for P_2_O_5_, 0.55 kg CO_2_-eq kg^-1^, for K_2_O, 2.66 kg CO_2_-eq mm^-1^ for irrigation water (electricity), and 55.4 kg CO_2_-eq ha^-^¹ for diesel fuel consumption during tillage and harvest (Chen et al., 2011; Zhang et al., 2017b).

Regarding the soil component ( $GWP_{soil}$), the calculation included cumulative CH4 and N2O emissions converted to CO2-equivalents over a 100-year horizon (with warming potentials of 28 and 265, respectively), as shown in Equation 10:

(10) $$\begin{array}{c} GWP_{soil}=28GF_{CH4}+265GF_{N2O} \end{array}$$

where $GWP_{soil}$ represents the GWP from direct soil fluxes (kg CO_2_-eq ha^-^¹). It is important to note that chamber-measured soil CO_2_ fluxes were excluded from the calculation. While soil respiration is a significant carbon flux, the static dark chamber method captures only ecosystem respiration (an output) without accounting for photosynthetic carbon uptake (an input). Including these fluxes would erroneously inflate the net carbon balance (Shang et al., 2019). Thus, our approach represents a conservative estimate focusing on non-CO_2_ gases and input-based emissions.

Greenhouse gas emission intensity (GHGI) was defined as the LCA-based GWP per unit of crop yield based on Equation 11 (IPCC, 2014):

(11) $$\begin{array}{c} GHGI=\frac{GWP}{Y} \end{array}$$

where *GHGI* is the GHG emission intensity (kg CO_2_-eq t^-^¹) and *Y* is the alfalfa hay yield (t ha^-^¹).

### 2.6 Statistical analysis

After data processing with Excel 2016 (Microsoft Corp., Redmond, WA, USA), statistical analyses were performed using IBM SPSS Statistics 20.0 (IBM Corp., Armonk, NY, USA). Two-way analysis of variance (ANOVA) followed by the least significant difference (LSD) test was used to identify differences in cumulative GHG emissions, crop yield, IWP, PFPN, GHGI, and GWP among treatments. Pearson correlation and linear regression were applied to assess relationships between GHG fluxes and soil properties. Graphs were generated using Origin 2023 (OriginLab Corp., Northampton, MA, USA). Prior to ANOVA, normality of residuals (Shapiro–Wilk test) and homogeneity of variances (Levene’s test) were examined. All variables met both assumptions (P > 0.05).

## 3 Results

### 3.1 Soil physicochemical dynamics in relation to GHG emissions

**Figure 3:**
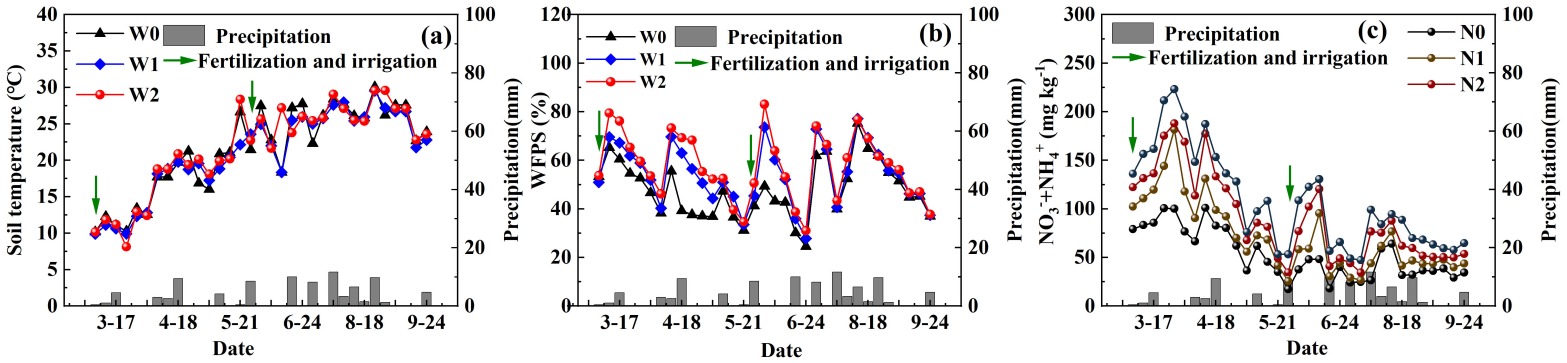
Temporal dynamics of soil physicochemical properties under different levels of irrigation (W0: 0 mm, W1: 100 mm, W2: 300 mm) and nitrogen application (N0: 0 kg ha^-^¹, N1: 60 kg ha^-^¹, N2: 120 kg ha^-^¹, N3: 180 kg ha^-^¹) during the alfalfa growing season. **(a)** Soil temperature at 0–5 cm depth, **(b)** Water-filled pore space (WFPS) at 0–20 cm depth, and **(c)** Soil available nitrogen content.

During the experimental period, the daily maximum and minimum temperatures in the study area were 39.9°C and –10.1°C, respectively, with the total precipitation reaching 578.7 mm. The average daily temperature over the alfalfa growing season ranged from –6.7°C to 31.6°C (**Figure 2**). Soil temperatures (0–5 cm) exhibited consistent trends across treatments and showed clear seasonal variation, primarily driven by air temperature and precipitation (**Figure 3a**). Under different irrigation treatments, the average soil temperature for W0 (21.35°C) was slightly, but not significantly, higher than that of W1 (20.94°C) and lower than that of W2 (21.64°C). WFPS varied prominently due to irrigation and rainfall, showing repeated wet-dry cycles. WFPS increased with higher irrigation volumes, ranging between 24.52–75.23% for W0, 27.82–77.08% for W1, and 31.02–83.06% for W2. Within each irrigation cycle, WFPS gradually decreased over time in similar patterns (**Figure 3b**). Under different nitrogen treatments, soil available nitrogen contents (0–20 cm) displayed comparable seasonal changes, with temporary increases followed by declines after irrigation or rainfall. The sum of soil NO_3_^–^ and NH_4_^+^ contents ranged from 13.7 to 232.18 mg kg^-^¹, and higher irrigation volumes or nitrogen rates maintained elevated soil nitrogen levels for longer durations. Soil available nitrogen contents trended downward over the alfalfa growing season (**Figure 3c**).

**Figure 4:**
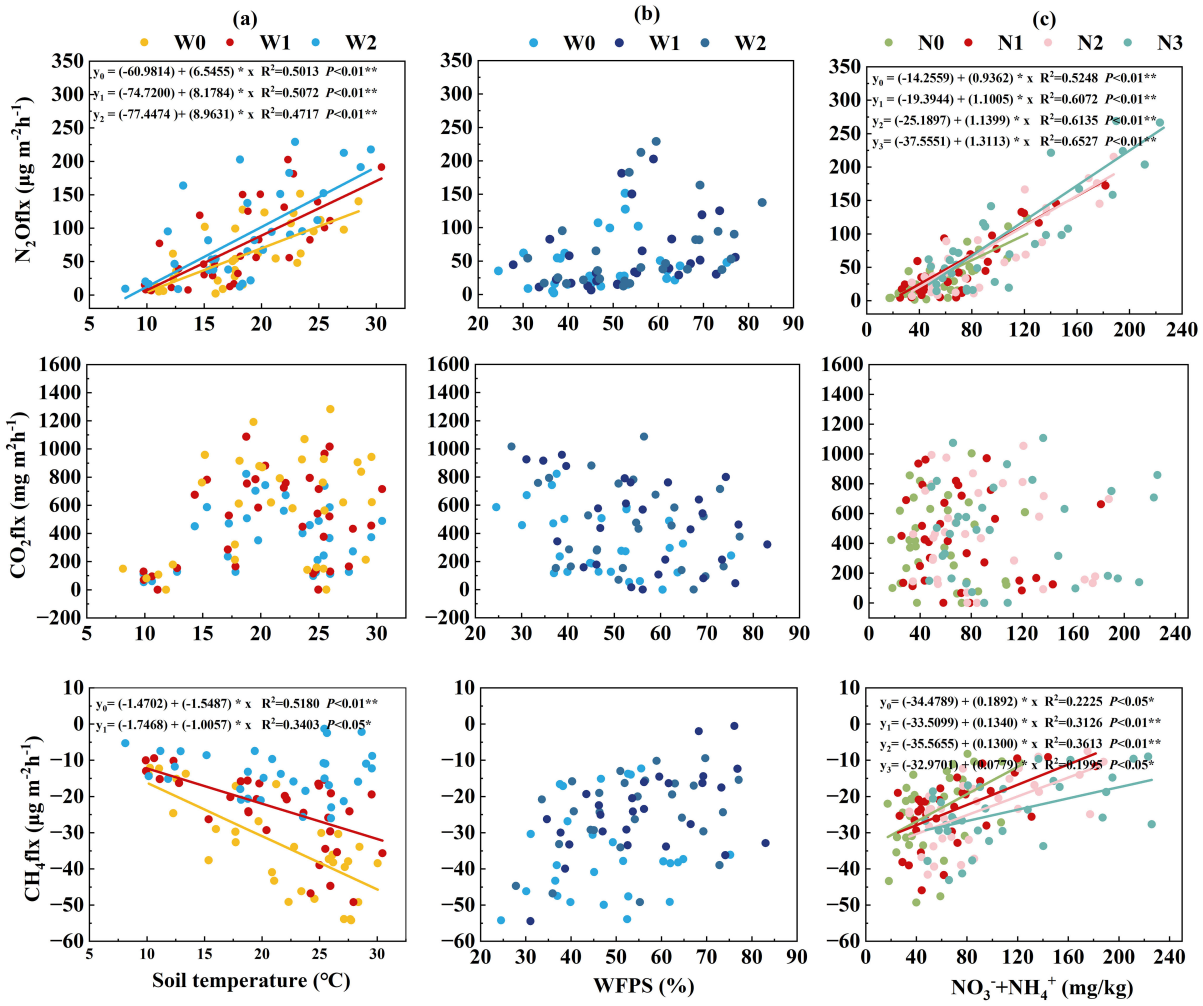
Relationship between greenhouse gas emission fluxes and **(a)** soil temperature at 0–5 cm depth, **(b)** soil available nitrogen content at 0–20 cm depth, and **(c)** water-filled pore space (WFPS). *P< 0.05, **P< 0.01, and ***P< 0.001; NS indicates no significance. Each data point represents the mean of three replicates for irrigation or nitrogen treatments. Only significant correlations (P< 0.05) are displayed; the complete panel is provided in **Supplementary Image 1**.

The relationships between GHG emission fluxes and soil physicochemical properties were assessed using correlation analysis. During the alfalfa growing season, N_2_O fluxes linearly increased with rising soil temperature and available nitrogen content, and higher fluxes were observed within the WFPS range of 50–60%. CO_2_ fluxes peaked at soil temperatures between 15°C–20°C and decreased linearly with increasing WFPS, with higher fluxes at WFPS of 35–45%. CH_4_ fluxes were negatively correlated with soil temperature and positively correlated with soil WFPS and available nitrogen content. Further, the relative contributions of soil physicochemical properties to N_2_O, CO_2_, and CH_4_ emission fluxes were assessed using linear regression. Regression equations were established for each gas emission flux against soil temperature, WFPS, and available nitrogen content. WFPS did not show significant linear correlations with N_2_O, CO_2_ or CH_4_ fluxes (P > 0.05; **Figure 4**), probably because soil moisture rarely reached the critical thresholds for sharp emission pulses under the semi-arid conditions of this study, leaving mineral N as the dominant short-term driver.

### 3.2 N_2_O fluxes and cumulative emissions

**Figure 5:**
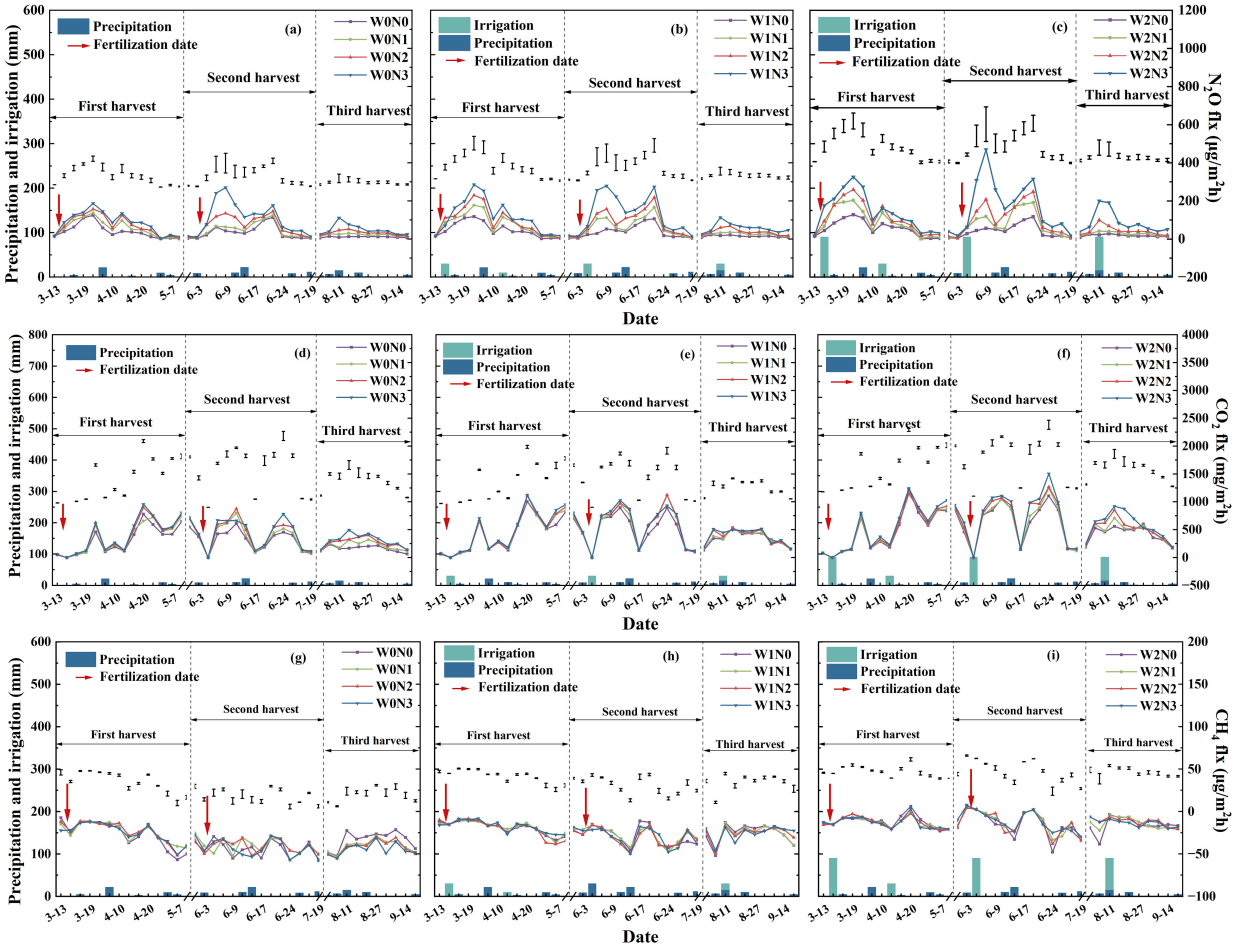
Greenhouse gas emission fluxes from unfertilized (N0) and nitrogen-fertilized (N1–N3) alfalfa fields under different irrigation treatments (W0: No irrigation, W1: Low water level, and W2: High water level). **(a–c)** N_2_O fluxes, **(d–f)** CO_2_ fluxes, and **(g–i)** CH_4_ fluxes.

N_2_O fluxes frequently rose within a week of N application or ≥10 mm rainfall and then subsided, with the largest pulses in W2N3 (**Figures 5a–c**). Because background variability was high and the routine sampling interval was 7–10 d, we confined our inference to treatment-level cumulative emissions rather than to exact lag times. W2 resulted in the greatest fluctuation in N_2_O fluxes over the alfalfa growing season (**Figure 5c**), followed by W1 (**Figure 5b**), and the smallest variation was observed for W0 (**Figure 5a**). Among the three harvest cycles, N_2_O fluxes were highest in the second cycle (1.09–469.35 μg m^-^² h^-^¹) compared to the first (1.09–326.24 μg m^-^² h^-^¹) and third (5.01–199.68 μg m^-^² h^-^¹) cycles under each irrigation level. In the third harvest cycle, N_2_O fluxes exhibited similar trends between W0 and W1, both of which were significantly lower than W2. Under different nitrogen treatments, N_2_O fluxes trended upward with higher nitrogen rates. In the third harvest cycle without topdressing, there were significantly lower N_2_O fluxes across all nitrogen treatments than in the first and second cycles. The average N_2_O flux peaked in W2N3, and was lowest in W0N0.

**Figure 6:**
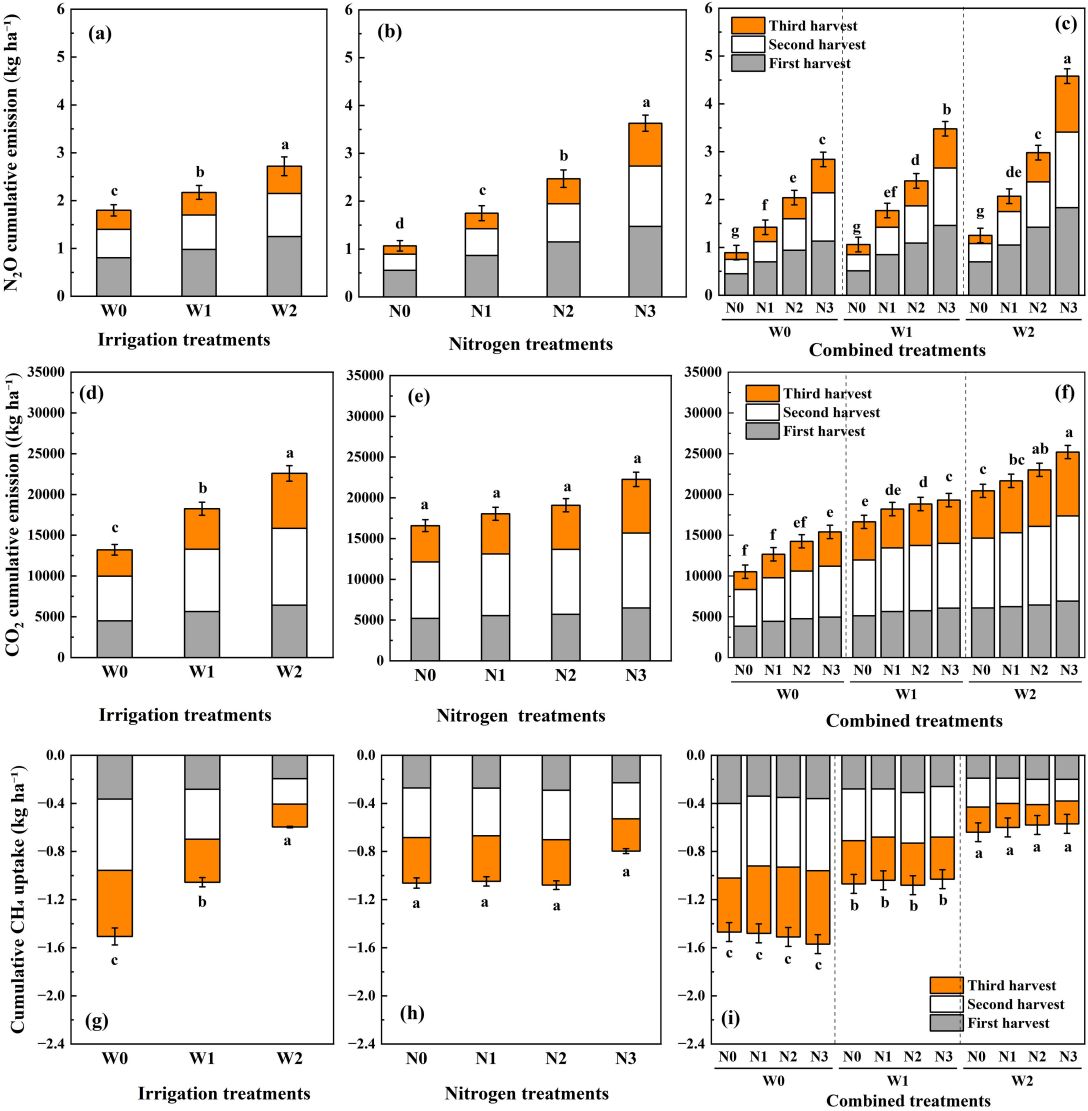
Effects of irrigation and nitrogen management on cumulative greenhouse gas emissions in alfalfa fields over three harvest cycles. **(a–c)** Cumulative N_2_O emissions, **(d–f)** cumulative CO_2_ emissions, and **(g–i)** cumulative CH_4_ uptake. The stacked bars represent the contributions of the first, second, and third harvests. Different lowercase letters above the bars indicate significant differences in the total annual cumulative emissions among treatments based on the LSD test (P< 0.05). Error bars represent the standard error of the means (n = 3). Treatments: W0 (0 mm), W1 (100 mm), W2 (300 mm); N0 (0 kg ha^-^¹), N1 (60 kg ha^-^¹), N2 (120 kg ha^-^¹), N3 (180 kg ha^-^¹).

Cumulative N_2_O emissions were significantly affected by fertilization and its interactions with irrigation (**Figures 6a–c**). Combined irrigation and nitrogen application significantly increased cumulative N_2_O emissions over the alfalfa growing season, and emissions increased proportionally with higher treatment levels. ANOVA results showed significant differences in cumulative N_2_O emissions among nitrogen treatments, but not among irrigation treatments (**Figures 6a, b**). During the three harvest cycles, cumulative N_2_O emissions decreased with lower nitrogen rates across all irrigation treatments and were lowest in the third cycle, with the highest in the first cycle. Cumulative N_2_O emissions peaked in W2 (2.72 kg ha^-^¹) and N3 (3.63 kg ha^-^¹) (**Figures 6a, b**). Among the combined treatments, increasing nitrogen rate under each irrigation level elevated cumulative N_2_O emissions. W2N3 produced the highest cumulative N_2_O emissions (4.59 kg ha^-^¹), with the lowest observed for W0N0 (0.89 kg ha^-^¹) (**Figure 6c**). These results indicate that post-fertilization N_2_O emissions were a major contributor to annual totals. In particular, emissions following basal and secondary nitrogen applications accounted for ~53% of the annual cumulative N_2_O emission.

### 3.3 CO_2_ fluxes and cumulative emissions

With regard to CO_2_ emission fluxes, there were significant effects of irrigation, fertilization, and their interactions (**Figures 5d–f**). CO_2_ fluxes showed consistent patterns across treatments, with W2 exceeding W1 and N1–N3 slightly higher than N0. Distinct temporal patterns emerged in CO_2_ fluxes under different water-nitrogen interactions. After fertilization and the first irrigation (March 14), maximum CO_2_ flux occurred within the first week of input, but equally rapid rises followed subsequent rains; thus, timing is co-controlled by temperature and moisture, not a fixed lag. Subsequent irrigation and rainfall events triggered new peaks in CO_2_ fluxes. After fertilization and the second irrigation (April 14), W0N0 maintained relatively stable CO_2_ fluxes, which peaked at 1033.76 mg m^-^² h^-^¹ on day 6 and then decreased afterwards. Following rainfall, CO_2_ fluxes remained persistently high for 0–6 days. Additionally, CO_2_ fluxes were temperature-controlled, generally lower in the cold months (November–March) than in the warm months (April–October).

Changes in cumulative CO_2_ emissions indicated significant effects of irrigation and irrigation-fertilization interactions (**Figures 6d–f**). ANOVA results revealed that cumulative CO_2_ emissions differed significantly among irrigation treatments, but not among nitrogen treatments (**Figures 6d, e**). Specifically, cumulative CO_2_ emissions increased with higher irrigation volumes, reaching 22,586.17 kg ha^-^¹ for W2. Within each irrigation treatment, cumulative CO_2_ emissions exhibited a mild upward trend with increasing nitrogen rate. The lowest annual cumulative CO_2_ emission was observed for W0N0 (10,530.18 kg ha^-^¹) and the highest for W2N3 (25,206.11 kg ha^-^¹) (**Figure 6f**). Emissions following irrigation and fertilization accounted for ~1.56% of the annual cumulative CO_2_ emissions across treatments.

### 3.4 CH_4_ fluxes and cumulative emissions

CH_4_ emission fluxes were significantly responsive to irrigation, but not to fertilization or their interactions (**Figures 5g–i**). All field plots consistently functioned as CH_4_ sinks throughout the experimental period. Soil CH_4_ uptake was transiently reduced after wetting, yet the response was inconsistent; only irrigation level significantly affected the seasonal sink strength. CH_4_ fluxes decreased with increasing irrigation volume, ranging from –77.95 μg m^-^² h^-^¹ (W0) to –8.14 μg m^-^² h^-^¹ (W2). No distinct temporal pattern in CH_4_ fluxes was observed across treatments. The effects of irrigation on cumulative CH_4_ uptake were also significant (**Figures 6g–i**). Cumulative CH_4_ uptake decreased with increasing irrigation volume, from 1.50 kg ha^-^¹ (W0) to 0.60 kg ha^-^¹ (W2) (**Figure 6h**). Emission peaks following irrigation and fertilization contributed ~1.02% to the annual cumulative CH_4_ emission.

### 3.5 Forage yield and resource use efficiency

**Figure 7:**
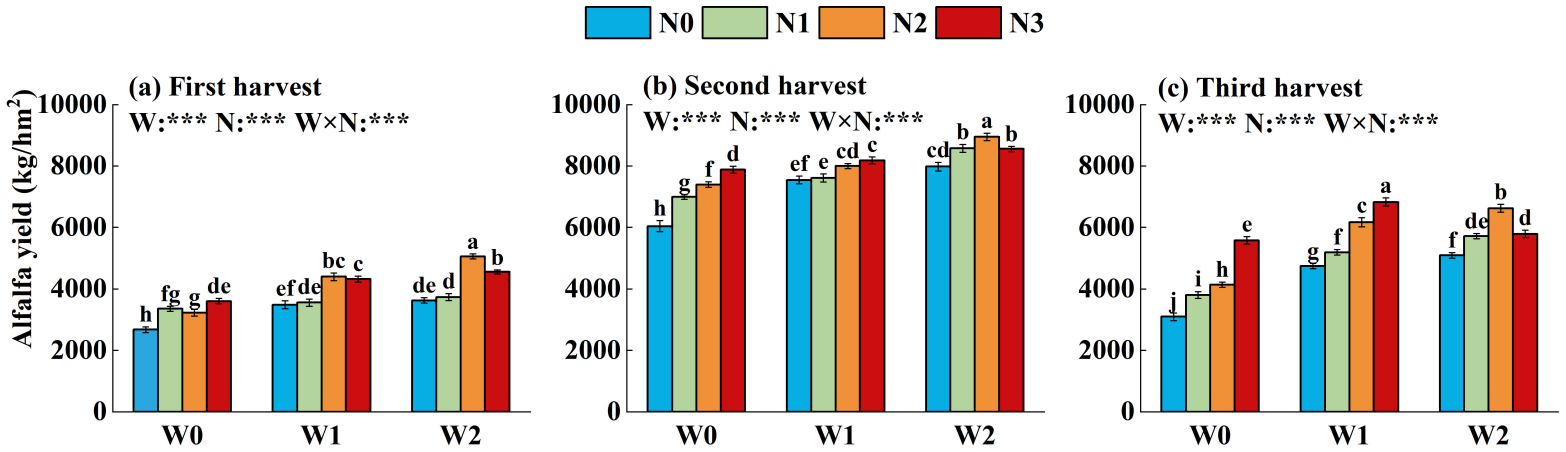
Effects of water-nitrogen treatments on alfalfa yield over three harvest cycles. **(a)** Yields in the first harvest cycle, **(b)** Yields in the second harvest cycle, and **(c)** Yields in the third harvest cycle. Significant differences between treatments are indicated by different lowercase letters above error bars. ***P< 0.001. W0: No irrigation, W1: Low water level, W2: High water level, N0: no fertilization, N1: Low nitrogen level, N2: Moderate nitrogen level, and N3: High nitrogen level.

Irrigation, fertilization, and their interactions all exhibited highly significant effects on alfalfa hay yield (**Figure 7**). Higher irrigation volumes led to improved alfalfa yields. Across all irrigation treatments, alfalfa yields increased initially and then decreased over successive harvest cycles. The mean yield was highest in the second harvest cycle (7,811.0 kg ha^-^¹) followed by the third cycle (5,232.4 kg ha^-^¹), and the lowest yield was recorded for the first cycle (3,799.1 kg ha^-^¹). Under W0 and W1 treatments, alfalfa yield increased with higher nitrogen rates, peaking at 6,069.46 kg ha^-^¹ for N3. Under W2 treatment, alfalfa yield peaked at 6,876.73 kg ha^-^¹ for N2 and then declined with higher nitrogen input; N3 resulted in lower alfalfa yield than N2 and had no significant difference compared to N1. The annual yield across irrigation-nitrogen treatments ranged from 18,907.30 kg ha^-^¹ (W2N2) to 11,811.79 kg ha^-^¹ (W0N0).

The effects of irrigation, fertilization, and their interactions on IWP were highly significant (**Table 2**). IWP was lower under W2 than under W1; however, this comparison is restricted to only two irrigated levels (W1 and W2) and should therefore be treated as indicative rather than a statistically robust trend. Across nitrogen treatments, IWP initially increased and then declined with increasing N rate. Among different nitrogen treatments, the highest IWP was observed for N2, exceeding that of N0 by 26.2% (W1) and 39.6% (W2). Under the combined treatments, IWP peaked in W1N3 (6.82 kg m^-^³), followed by W1N2 (6.67 kg m^-^³) in the second harvest cycle. W2N2 improved IWP by 10.9% compared to the conventional high-water, high-nitrogen regime W2N3.

**Table 2:** Effects of water-nitrogen treatments on irrigation water productivity (IWP) and partial factor productivity of nitrogen (PFPN) in alfalfa fields.

Irrigation treatment	Nitrogen treatment	IWP (kg m^-^³)			PFPN (kg kg^-^¹)
		First harvest	Second harvest	Third harvest	
W0	N0	–	–	–	–
	N1	–	–	–	235.92 ± 4.38c
	N2	–	–	–	122.99 ± 2.38f
	N3	–	–	–	94.87 ± 1.74h
W1	N0	2.90 ± 0.11b	6.29 ± 0.10c	3.95 ± 0.08d	–
	N1	2.96 ± 0.10b	6.35 ± 0.11c	4.33 ± 0.07c	272.65 ± 5.55b
	N2	3.66 ± 0.10a	6.67 ± 0.06b	5.14 ± 0.12b	154.73 ± 2.91e
	N3	3.60 ± 0.09a	6.82 ± 0.10a	5.70 ± 0.11a	107.44 ± 1.88g
W2	N0	1.01 ± 0.02e	2.22 ± 0.04e	1.41 ± 0.02g	–
	N1	1.03 ± 0.03e	2.38 ± 0.03d	1.59 ± 0.02f	300.55 ± 5.44a
	N2	1.41 ± 0.02c	2.49 ± 0.04d	1.84 ± 0.03e	171.92 ± 2.78d
	N3	1.27 ± 0.02d	2.37 ± 0.02d	1.61 ± 0.03f	105.04 ± 1.48g
Water		***	***	***	***
Nitrogen		***	***	***	***
W × N		***	***	***	***

Data are means ± standard error (n = 3). Different lowercase letters within the same column indicate significant differences between treatments. ***P < 0.001.

The PFPN patterns also indicated highly significant effects of irrigation, fertilization, and their interactions (**Table 2**). PFPN increased with higher irrigation volumes, while it negatively responded to nitrogen rate under each irrigation level. Among all combined treatments, the highest PFPN was recorded for W2N1 (300.55 kg kg^-^¹), and the lowest for W0N3 (94.87 kg kg^-^¹). W2N2 achieved a favorable balance between alfalfa yield and nitrogen use efficiency, improving PFPN by 56.9% compared to W2N3.

### 3.6 Climate response to GHG emissions

To assess the climate response to GHG emissions in alfalfa fields under water-nitrogen management, ANOVA was conducted for EF, GWP, and GHGI across treatments. All three parameters showed significant differences in relation to irrigation, fertilization, and their interactions (**Table 3**). EF showed an upward trend with increasing irrigation volume. Under both W0 and W2 levels, EF rose with higher nitrogen rates. Under the W1 level, N2 produced the lowest EF (1.10%). The highest EF value (1.86%) was observed for W2N3, which also recorded the greatest GWP and GHGI values under combined treatments. When incorporating indirect emissions from agricultural inputs, the total GWP for W2N3 reached 3721.54 kg CO_2_-eq ha^-^¹. Compared to W2N3, W2N2 achieved favorable trade-offs, reducing EF (by 29.1%),total GWP (by 24.1%), and GHGI (by 29.0%). This reduction was primarily driven by lower nitrogen fertilizer inputs, mitigating both direct soil emissions and upstream industrial carbon costs. In summary, soil GHG emissions showed a strong positive response to high water and nitrogen inputs, while the high-water, medium-nitrogen regime effectively reduced GHG emissions from alfalfa production. The highest N_2_O emission factor (1.86%) under W2N3 approached twice the IPCC default (1.0%), while optimized treatments (≤ 120 kg N ha^-^¹) fell within ranges reported for alfalfa systems (0.4–1.7%; Fernández-Ortega et al., 2024; Kamran et al., 2022).

**Table 3:** Effects of water-nitrogen treatments on N_2_O emission factor (EF), total global warming potential (GWP), and greenhouse gas emission intensity (GHGI) (LCA-based).

Treatment		EF (%)	GWP (kg CO_2_-eq ha^-^¹)			GHGI (kg CO_2_-eq t^-1^)		
			First harvest	Second harvest	Third harvest	First harvest	Second harvest	Third harvest
W0	N0	–	250.41 ± 2.80 l	89.96 ± 0.71 l	50.36 ± 2.28 l	93.73 ± 1.65 l	14.89 ± 0.58 j	16.27 ± 0.49 j
	N1	0.87	675.13 ± 13.17 i	281.06 ± 15.82 i	95.24 ± 3.44 k	201.07 ± 9.29 i	40.20 ± 5.84 h	25.03 ± 0.43 h
	N2	0.96	1095.72 ± 2.44 g	497.69 ± 5.20 g	134.33 ± 4.94 j	339.73 ± 9.09 d	67.29 ± 1.27 f	32.47 ± 0.93 f
	N3	1.07	1497.71 ± 9.02 d	748.41 ± 5.58 d	208.08 ± 3.60 f	415.65 ± 21.09 b	94.90 ± 3.11 c	37.27 ± 0.79 e
W1	N0	–	379.26 ± 1.17 k	189.05 ± 5.01 j	149.80 ± 0.91 i	109.07 ± 4.59 k	25.06 ± 1.63 i	31.58 ± 0.35 f
	N1	1.17	826.17 ± 7.13 h	404.76 ± 5.83 h	193.78 ± 2.90 g	232.59 ± 4.03 h	53.20 ± 2.84 g	37.34 ± 1.60 e
	N2	1.10	1246.94 ± 5.03 e	616.32 ± 7.73 e	243.55 ± 5.27 d	283.56 ± 5.96 g	77.03 ± 2.05 e	39.47 ± 0.72 d
	N3	1.34	1707.59 ± 8.33 b	883.49 ± 9.94 b	331.94 ± 3.06 b	395.03 ± 6.22 c	107.97 ± 3.12 b	48.59 ± 0.37 c
W2	N0	–	648.73 ± 3.47 j	363.30 ± 4.46 h	300.72 ± 2.43 c	179.05 ± 3.18 j	45.55 ± 1.28 h	59.05 ± 3.72 b
N1	1.36	1101.53 ± 0.80 f	607.41 ± 10.23 f	344.54 ± 3.94 b	295.10 ± 8.59 f	70.77 ± 2.91 f	60.25 ± 3.50 b
N2	1.44	1559.97 ± 0.68 c	831.82 ± 6.70 c	431.41 ± 3.60 a	308.57 ± 4.68 e	92.94 ± 1.90 d	65.17 ± 2.54 a
N3	1.86	1979.94 ± 61.36 a	1173.25 ± 9.31 a	568.35 ± 13.80 a	434.93 ± 60.30 a	137.10 ± 1.61 a	98.08 ± 2.51 a
Water			***	***	***	***	***	***
Nitrogen			***	***	***	***	***	***
W× N			**	***	***	**	***	***

Data are means ± standard error (*n* = 3). Different lowercase letters within the same column indicate significant differences among treatments based on the LSD test at P< 0.05. Asterisks indicate the significance levels of the analysis of variance (ANOVA): * P< 0.05, ** P< 0.01, and *** P< 0.001; NS indicates no significance. GWP and GHGI values include both direct soil emissions and indirect emissions from agricultural inputs (LCA-based). Percentage reductions of GWP and GHGI mentioned in the text are calculated relative to the conventional high-input reference W2N3 (300 mm irrigation + 180 kg N ha^-1^); EF is computed against the corresponding N0 treatment.

## 4 Discussion

### 4.1 Enhancement of soil N_2_O emission fluxes by water-nitrogen interactions

N_2_O is produced mainly through microbial nitrification and denitrification processes, and soil N_2_O emissions are highly sensitive to soil moisture content, temperature, and nitrogen availability (Qian et al., 2025). Irrigation can indirectly promote N_2_O emission fluxes. In the absence of nitrogen application, increased irrigation levels had a limited effect on soil N_2_O emissions from alfalfa fields. However, when nitrogen was applied, irrigation markedly enhanced N_2_O emissions, possibly because increased soil moisture content indirectly contributed to N_2_O emissions by improving NO_3_^–^N availability (Ullah et al., 2016). It has been reported that elevated WFPS levels are associated with enhanced soil denitrification (Anderson et al., 2019). N_2_O emission fluxes were promoted by soil WFPS, as indicated by their significant positive correlation observed in this study. The underlying mechanism is that high irrigation levels (WFPS 50–60%) facilitated soil denitrification. This finding is consistent with the denitrification thresholds reported by Bracken et al. (2021) and Wang et al. (2023).

The application of nitrogen fertilizer resulted in considerably higher soil N_2_O emissions in alfalfa fields, as nitrogen application elevated soil available nitrogen content, providing ample substrates for nitrifying and denitrifying bacteria (Xun et al., 2022). N_2_O emission fluxes were positively driven by soil available nitrogen content, based on the results of correlation and regression analyses. There were stepwise rises in N_2_O fluxes from low nitrogen (N1) to high nitrogen (N3) treatments compared to the unfertilized control (N0), and the fluxes had strong positive correlation with soil available nitrogen content that surged immediately after fertilization. NO_3_^–^N is considered the primary source of nitrogen for N_2_O emissions. Excessive nitrogen not absorbed by alfalfa roots remains in the soil, serving as substrates for microbial nitrification and denitrification, consequently stimulating N_2_O emissions (Jiang et al., 2025; Wang et al., 2024). These findings emphasize the importance of optimizing irrigation and nitrogen rates to control soil N_2_O emissions from alfalfa cultivation. Given the 7–10 d routine sampling interval, we confine our interpretation to treatment-level cumulative emissions (**Figures 6a–c**) rather than to exact lag times.

### 4.2 Effects of water-nitrogen interactions on soil CO_2_ flux and emission dynamics

Soil CO_2_ fluxes in alfalfa fields reflected combined microbial and root respiration, with a smaller contribution from carbonate dissolution (Bai et al., 2021). Nitrogen fertilization stimulated microbial mineralization and root metabolic activity, thereby increasing CO_2_ release during N transformation processes (Hauck, 1981; Snyder et al., 2009; Kong et al., 2020). Short-term CO_2_ pulses appeared immediately after irrigation and fertilization, consistent with observations under fixed-moisture conditions (Pan et al., 2023). However, the strength of these responses differed among growth stages, indicating that water-nitrogen (W-N) interactions do not exert a uniform influence throughout the season.

The interaction was strongest during early regrowth. Following irrigation, CO_2_ fluxes increased sharply, reflecting the rewetting-induced activation of soil microbes documented in dryland systems (Zhai et al., 2011; Badaou and Sahin, 2025). These pulses overlapped with nitrogen addition, which provided readily available substrates for microbial decomposition and root respiration, producing higher peaks than those reported in single-factor experiments (Pan et al., 2023). The highest fluxes occurred on days 7 and 6 after irrigation, highlighting the system’s sensitivity to combined moisture and nutrient inputs. During peak biomass, CO_2_ fluxes were driven mainly by temperature and sustained soil moisture, whereas nitrogen effects weakened. Irrigation maintained higher soil moisture and supported root and microbial respiration, resulting in notably greater cumulative CO_2_ emissions under W2 than W0. This pattern aligns with evidence that adequate moisture enhances autotrophic and heterotrophic respiration when plant growth is vigorous (Wang et al., 2022). After harvest, CO_2_ fluxes declined and W-N interactions were minimal. Reduced root activity and lower belowground carbon allocation (Kong et al., 2020) diminished nitrogen-induced differences, while irrigation produced only moderate variation through soil moisture regulation.

Overall, W-N interactions had their strongest effects immediately after irrigation during early regrowth, whereas temperature and sustained moisture largely controlled cumulative CO_2_ emissions. This stage-dependent behavior explains the varying responses among harvest periods and underscores the combined influence of moisture pulses and plant phenology on CO_2_ dynamics in semi-arid alfalfa systems.

### 4.3 Functional stability of soil CH_4_ sinks under water-nitrogen regimes

CH_4_ is produced by methanogenic archaea through anaerobic decomposition of soil organic matter. Under aerobic conditions, methanotrophic microorganisms consume CH_4_ as an energy source along with O_2_. Therefore, the soil functions as a CH_4_ source when CH_4_ production by methanogens exceeds its oxidation by methanotrophs, and shifts to a CH_4_ sink when methanotrophs become dominant (Angle et al., 2017). During alfalfa cultivation, different water-nitrogen regimes did not significantly affect CH_4_ fluxes, supporting the results from earlier pot and field experiments showing that alfalfa soils consistently act as CH_4_ sinks (Fernández-Ortega et al., 2024; Kamran et al., 2022; Ning et al., 2020). However, previous studies collected monthly data from static chamber measurements or considered only one irrigation level, which contrasts with our high-frequency (daily) measurements across three irrigation and four nitrogen levels. The results showed that even pronounced wet-dry cycles and nitrogen pulses did not stimulate CH_4_ uptake or cause net emissions, highlighting the robustness of the soil sink function under intensive management. Based on the positive correlation between soil CH_4_ emission fluxes and WFPS, high WFPS levels under intensive irrigation likely improved soil porosity and structure, creating anaerobic conditions favorable for methanogenic activity. Similarly, Chen et al. (2021) reported that high soil moisture content reduced O_2_ availability, expanding anaerobic zones and enhancing CH_4_ production by methanogens. Although WFPS showed no significant linear correlation with CH_4_ fluxes (**Figure 4**), non-linear or threshold moisture effects may exist; these could not be tested with the present single-year dataset, so only significant linear relationships are retained in **Figure 4** and the complete matrix is provided in **Supplementary Image 1**.

The effect of nitrogen application on CH_4_ emissions remains inconclusive. Nitrogen rate did not significantly affect CH_4_ emissions from alfalfa production, although findings from other crops may offer useful insights. For example, nitrogen fertilization has been identified as a major factor affecting CH_4_ emissions in cotton systems (Bai et al., 2021). In wheat systems, moderate nitrogen levels may enhance CH_4_ oxidation, while excessive fertilization or topdressing can inhibit methanotrophic activity, resulting in increased CH_4_ emissions (Hütsch et al., 1993).

### 4.4 Trade-offs, synergies, and resource use efficiencies of water and nitrogen

Irrigation and fertilization play essential roles in achieving high yield and quality of alfalfa in arid and semi-arid regions (Ma et al., 2024). While nitrogen application increased annual alfalfa yield compared to the control, there was a non-linear yield response to nitrogen rate. This pattern is attributed to the role of nitrogen as a key determinant of crop productivity. When applied at optimal levels, nitrogen improves alfalfa yield by promoting cell division and growth and enhancing dry matter accumulation (Lv et al., 2025; Ma et al., 2024). However, excessive nitrogen input may suppress rhizobial nitrogen fixation, reducing nitrogen use efficiency (Hu et al., 2019). Our results indicated that high nitrogen application (N3) did not improve alfalfa yield, potentially due to reduced nitrogen use efficiency and increased GHG emissions. Additionally, alfalfa yield increased progressively with higher irrigation levels, consistent with previous findings by Jia et al. (2018) under a single nitrogen level. The rate of yield increase was amplified under moderate nitrogen application and peaked when the nitrogen level reached 120 kg ha^-^¹. During the alfalfa growing season, substantial rainfall in June–August 2024 provided sufficient soil moisture, leading to the highest crop yield in the second harvest. It has been suggested that moderately increasing fertilization and irrigation promotes improvements in fresh forage yield, while over-fertilization with poor irrigation management can lead to yield reduction (Kandelous et al., 2012). No yield reduction due to irrigation was observed in the present study, possibly because the applied water volumes did not exceed the critical threshold. Within a suitable soil moisture range, appropriate fertilization notably improved alfalfa productivity (Mu et al., 2025).

IWP and PFPN are key indicators for evaluating water and fertilizer management in agricultural systems, and both of them are strongly affected by irrigation and fertilization strategies. Wen et al. (2024) suggested that IWP increases with higher irrigation volumes within a specific range, whereas Djaman et al. (2020) reported the opposite pattern. In this study, IWP decreased as irrigation volume increased, and increasing nitrogen application improved IWP under each irrigation level, suggesting a synergistic interaction between water and fertilizer inputs. Appropriate fertilization can promote root growth and distribution, enhancing water and nutrient uptake, thereby improving IWP under water-limited conditions. Additionally, it has been shown that PFPN increases first and then declines with increasing nitrogen rate over a reasonable range (Mu et al., 2025). This suggests that moderate nitrogen input enhances crop yield, whereas excessive nitrogen application causes fertilizer loss and environmental risk. Results of this study showed that PFPN declined with increasing nitrogen rate across all irrigation levels, indicating that excessive nitrogen input did not necessarily improve fertilizer use efficiency. Coordinated water and fertilizer management during alfalfa cultivation achieved the dual goals of “managing water through fertilizer” and “enhancing fertilizer use efficiency through water.” The water-fertilizer synergy was maximized under combined application of appropriate irrigation and nitrogen levels, effectively improving alfalfa yield and resource use efficiency. For example, both PFPN and IWP peaked under moderate nitrogen application (180 kg ha^-^¹) with high irrigation (300 mm).

### 4.5 Optimizing water and nitrogen inputs to mitigate GWP and GHGI

Soil GHG emissions remained positive across all irrigation and nitrogen treatments, indicating that the alfalfa system acted as a net GHG source. GWP increased under higher irrigation levels with elevated nitrogen rates, suggesting that both water and nitrogen were key drivers influencing this metric. Specifically, the high GWP in intensive treatments was driven not only by soil denitrification but also by the substantial carbon footprint embedded in agricultural inputs. N_2_O and CH_4_ are recognized as the two most important soil-derived GHGs in GWP assessments (Li et al., 2023), and their emissions are highly sensitive to both irrigation and fertilization practices. Under arid soil conditions, CH_4_ uptake often exceeds its emissions, making N_2_O the dominant contributor to soil-based GWP in alfalfa systems (Zhang et al., 2024). Fertilizer application can promote microbial activity in the soil. For example, NH_4_^+^-based nitrogen fertilizer provides readily available substrate for ammonia-oxidizing bacteria, stimulating soil nitrification (Fudjoe et al., 2023). However, excessive application of basal or topdressing fertilizer disrupts the balance between soil nitrogen supply and plant uptake. This could result in residual mineral nitrogen and elevated N_2_O emissions, consequently increasing soil GWP.

Although this study integrated LCA to calculate comprehensive GWP, it should be noted that excluding soil CO_2_ introduces uncertainty because chamber-based CO_2_ respiration does not represent net ecosystem carbon exchange. Therefore, the GWP and GHGI values reported here reflect only direct non-CO_2_ soil fluxes and indirect input emissions, explicitly excluding chamber-measured soil respiration. Future studies should combine chamber measurements with ecosystem-scale carbon assessments for a more complete evaluation. This approach aligns with protocols focusing on non-CO_2_ greenhouse gases in short-term studies (Shang et al., 2019; Smith et al., 2020).

GHGI is a key metric for evaluating agricultural sustainability. Optimal fertilization can enhance alfalfa yield while lowering GHGI (Ma et al., 2024). This study showed that moderate nitrogen application (N2) resulted in higher alfalfa yield (by 1.15–7.02%) and lower LCA-based GHGI (by ~29.0%) compared to high nitrogen treatment (N3). The reduction in GHGI for N2 was due to the dual mitigation of direct soil N_2_O emissions and the indirect carbon footprint from fertilizer production. Our LCA analysis revealed that ‘hidden’ carbon emissions from inputs are major drivers of GWP; optimizing the N rate to 120 kg ha^-1^ (W2N2) effectively cut these upstream emissions by approximately 33% compared to the N3 level. Therefore, alleviating GHGI in agricultural systems requires optimized nitrogen management to lower GHG emissions and improve crop yields—dual objectives that can be simultaneously achieved by adjusting the fertilization rate. Additionally, rational irrigation management can reduce nutrient leaching and GHG emissions associated with excessive water use (Wang et al., 2025).

Aligning irrigation with crop water demand is helpful to minimize GHGI while sustaining crop productivity. Conventional practices involving high water and nitrogen inputs were associated with elevated GHGI, whereas the optimal treatment (W2N2) reduced environmental impacts, resulting in lower GHGI. This study covered only one growing season at a single site; both weather conditions and GHG emissions are known to exhibit substantial inter-annual variability. Consequently, the proposed water–nitrogen management recommendations should be viewed as site-specific and year-specific, rather than universally optimized thresholds. Multi-year, multi-location experiments are needed to test the stability and generality of the observed synergy zone.

## 5 Conclusions

This study explored GHG emission patterns and yield responses of alfalfa in an arid and semi-arid region under varying water and nitrogen regimes. The results demonstrated significant irrigation–nitrogen interactions affecting soil N_2_O and CO_2_ fluxes, with CH_4_ uptake remaining relatively stable. N_2_O emissions were primarily driven by nitrogen input and modulated by soil moisture fluctuations, while CO_2_ fluxes were influenced mainly by irrigation.

A high irrigation regime (300 mm) combined with a moderate nitrogen rate (120 kg ha^-^¹) maintained high yield while substantially reducing cumulative GHG emissions and emission intensity. Specifically, the W2N2 treatment achieved approximately 24.1% lower total GWP (LCA-based) and 29.0% lower emission intensity than the high-water, high-nitrogen combination (W2N3), indicating improved resource-use efficiency and environmental performance. In contrast, excessive nitrogen or insufficient irrigation caused either elevated emissions or reduced productivity.

Overall, the present single-year, single-site results suggest a possible site- and year-specific synergy region (approximately 300 mm irrigation and 120 kg ha^-^¹), which performed well in balancing yield, resource efficiency, and greenhouse gas emissions. However, because interannual variations in precipitation, temperature, and soil microbial activity can substantially influence GHG fluxes, this recommended zone should be regarded as preliminary and requires multi-year validation before being generalized to wider regions.

Despite these limitations, our findings provide several practical management implications for semi-arid alfalfa production: (1) Avoid excessive nitrogen (> 120 kg ha^-^¹) under high irrigation, as this combination sharply increases N_2_O emissions without proportional yield gain. (2) A moderate nitrogen rate of ≈ 120 kg ha^-^¹ under adequate water (≈ 300 mm) simultaneously improves partial-factor productivity of nitrogen (PFPN) and maintains high biomass. (3) Under water-limited conditions, optimizing irrigation timing contributes more to irrigation-water productivity (IWP) than simply increasing nitrogen inputs.

## Data Availability

The original contributions presented in the study are included in the article/**Supplementary Material**. Further inquiries can be directed to the corresponding author/s.
