# Supplementary figures and images for: Interactive effects of irrigation and nitrogen management on greenhouse gas emissions and resource efficiency in alfalfa production
Source: Front Plant Sci. 2026 Jan 22;16:1740107. doi: 10.3389/fpls.2025.1740107 (PMC12875387; doi:10.3389/fpls.2025.1740107)

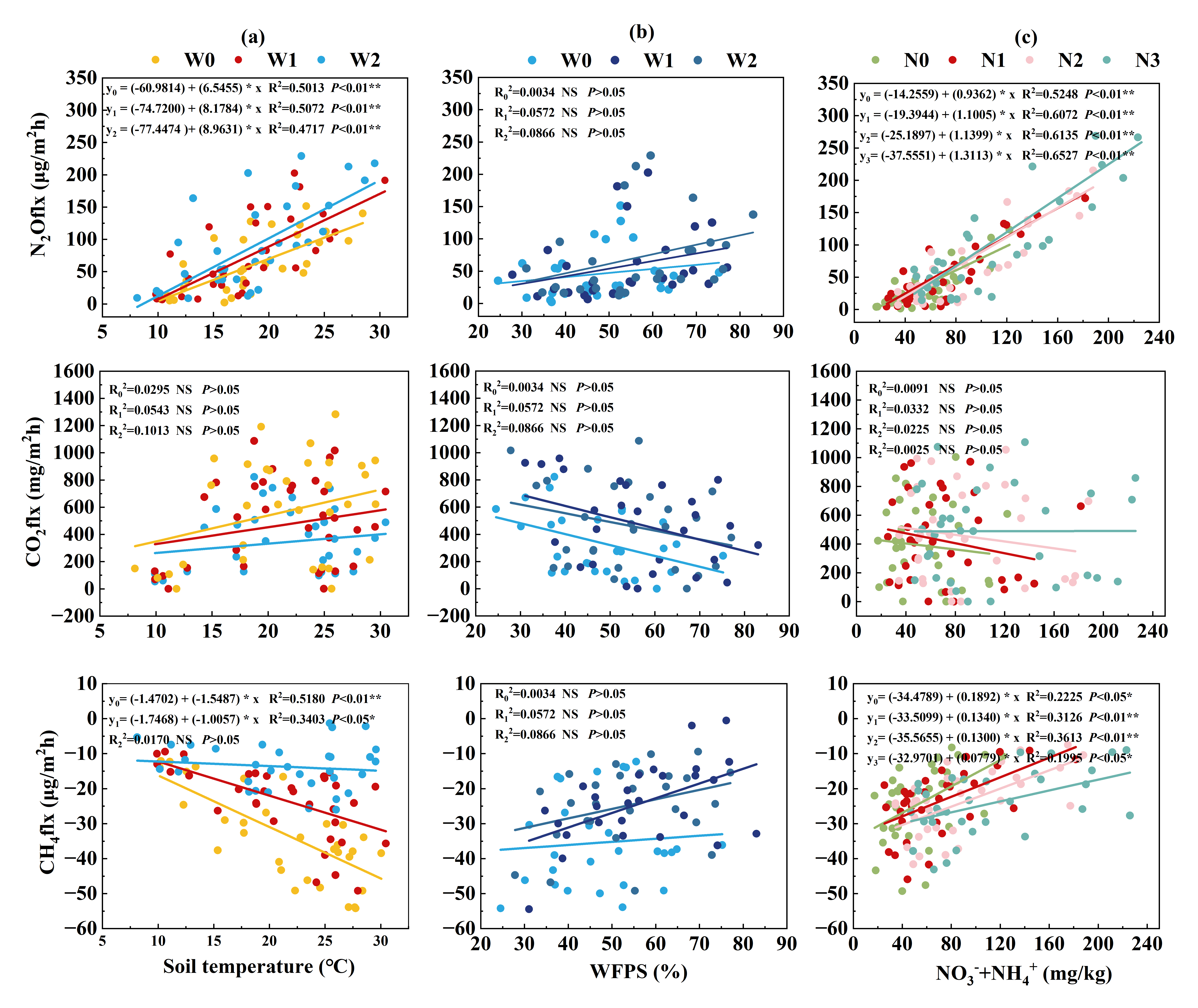

Supplement: Supplementary file 1 [file Image1.jpg]
